# AMPK-dependent phosphorylation of cingulin reversibly regulates its binding to actin filaments and microtubules

**DOI:** 10.1038/s41598-018-33418-7

**Published:** 2018-10-19

**Authors:** Tomoki Yano, Takayuki Torisawa, Kazuhiro Oiwa, Sachiko Tsukita

**Affiliations:** 1 Laboratory of Biological Science, Graduate School of Frontier Biosciences and Graduate School of Medicine, Osaka University, Osaka, 565-0871 Japan; 2 National Institute of Information and Communications Technology, Advanced ICT Research Institute, Kobe, Hyogo, 651-2492 Japan; 3 Present Address: Department of Genetics, School of Life Science, SOKENDAI (The Graduate University for Advanced Studies), Mishima 411-8540 Japan

## Abstract

Cytoskeletal organization is essential for the precise morphogenesis of cells, tissues, and organs. Cytoskeletons, bound to scaffolding proteins, regulate the apical junction complex (AJC), which is composed of tight and adherens junctions, and located at the apical side of epithelial cell sheets. Cingulin is a tight junction-associated protein that binds to both actin filaments and microtubules. However, how cingulin binds to microtubules and whether cingulin can bind to actin and microtubules simultaneously are unclear. Here we examined the mechanisms behind cingulin’s cytoskeleton-binding properties. First, using total internal reflection fluorescence microscopy, we detected cingulin at microtubule cross points. We then found the interdomain interactions in cingulin molecules. Notably, we found that this interaction was regulated by AMPK-dependent phosphorylation and changed cingulin’s conformation and binding properties to actin filaments and microtubules. Finally, we found that the AMPK-regulated cingulin properties regulated the barrier functions of epithelial cell sheets. We propose that the cellular metabolic state, which involves AMPK, can contribute to the organization and maintenance of epithelial tissues through cingulin’s tight junction/cytoskeleton regulation.

## Introduction

Epithelial cell sheets compartmentalize our body, with their apical membranes facing outer environments. To adapt to the outer environment and maintain inner homeostasis, apical membranes develop special architectures that include cytoskeletons^1–5^. For example, actin filaments, a type of cytoskeleton, located beneath the cell membrane, participate in the apical formation of microvilli, terminal webs, and circumferential ring organizations^2,6–9^. The circumferential ring is linked to the apical junctional complex (AJC), which is composed of the tight junction (TJ) and the adherens junction (AJ), and the TJ and AJ participate in the AJC function^10,11^. In addition to actin filaments, networks of microtubules and intermediate filaments also exist beneath the apical membrane of epithelial cells and appear to be associated with the AJC^4,12–16^.

Cytoskeletal interactions mediated by cross-linking proteins play essential roles in various biological events^4,17–19^. The interaction between actin filaments and microtubules was first described in a report showing that microtubules are prerequisite for the behavior of actin-rich protrusions at the leading edge of a migrating fibroblast^20^. Since that report, microtubule-actin filament interactions have been found to play fundamental roles across species from yeast to humans^19,21,22^. Many other proteins involved in this interaction have also been reported^19,23^; however, it remains largely unclear whether such proteins prefer to bind actin filaments, microtubules, or both simultaneously.

Cingulin, a TJ protein that forms a complex with one of the zonula occludens proteins (ZO-1, 2, or 3), is an a classical actin-binding protein whose function has been explored^24–28^. Cingulin is composed of three domains: a globular head, a coiled-coil rod, and a small globular tail^29^. The N-terminal globular head domain of cingulin has a high binding affinity for ZO proteins and actin filaments^26,27^. On the other hand, we previously reported that cingulin binds to microtubules and is phosphorylated in its head domain by AMP-activated protein kinase (AMPK)^14^. Recent studies have shown that cingulin directly binds to the C-terminal domain of tubulin^29^. However, the dynamic aspects of cingulin’s binding to actin filaments and microtubules, and the relative binding affinities of cingulin to actin filaments and microtubules remain unclear.

We started investigating the mechanisms behind these processes with the hypothesis that AMPK is responsible for the phosphorylation and conformational changes of cingulin, which affect its binding to microtubules and actin filaments. Here we examined these mechanisms using *in vitro* reconstitution experiments. At first, we showed by total internal reflection fluorescence (TIRF) microscopy that cingulin localizes to the sites where two microtubules intersect, but not to their plus or minus ends. This binding may be involved in the interactions between TJs and the sides of microtubules. In addition, by biochemical experiments and electron microscopy observations, we found that the N-terminal cingulin head interacts with its C-terminal domain. This interaction causes a conformational change in the cingulin molecule and is associated with its dephosphorylated state. We found that cingulin’s phosphorylation by AMPK positively regulates its interaction with microtubules. We discovered that the phosphorylated cingulin head domain tends to detach from actin filaments, and this tendency effects on the TJ’s permselectivity and sealing properties. Since AMPK is a critical enzyme in cellular energy metabolism, these findings provide new information about how the TJ function is controlled by the energy state of cells through modification of the TJ-cytoskeletal interaction.

## Results

### Cingulin is a cross-linker of microtubules

**Figure 1:**
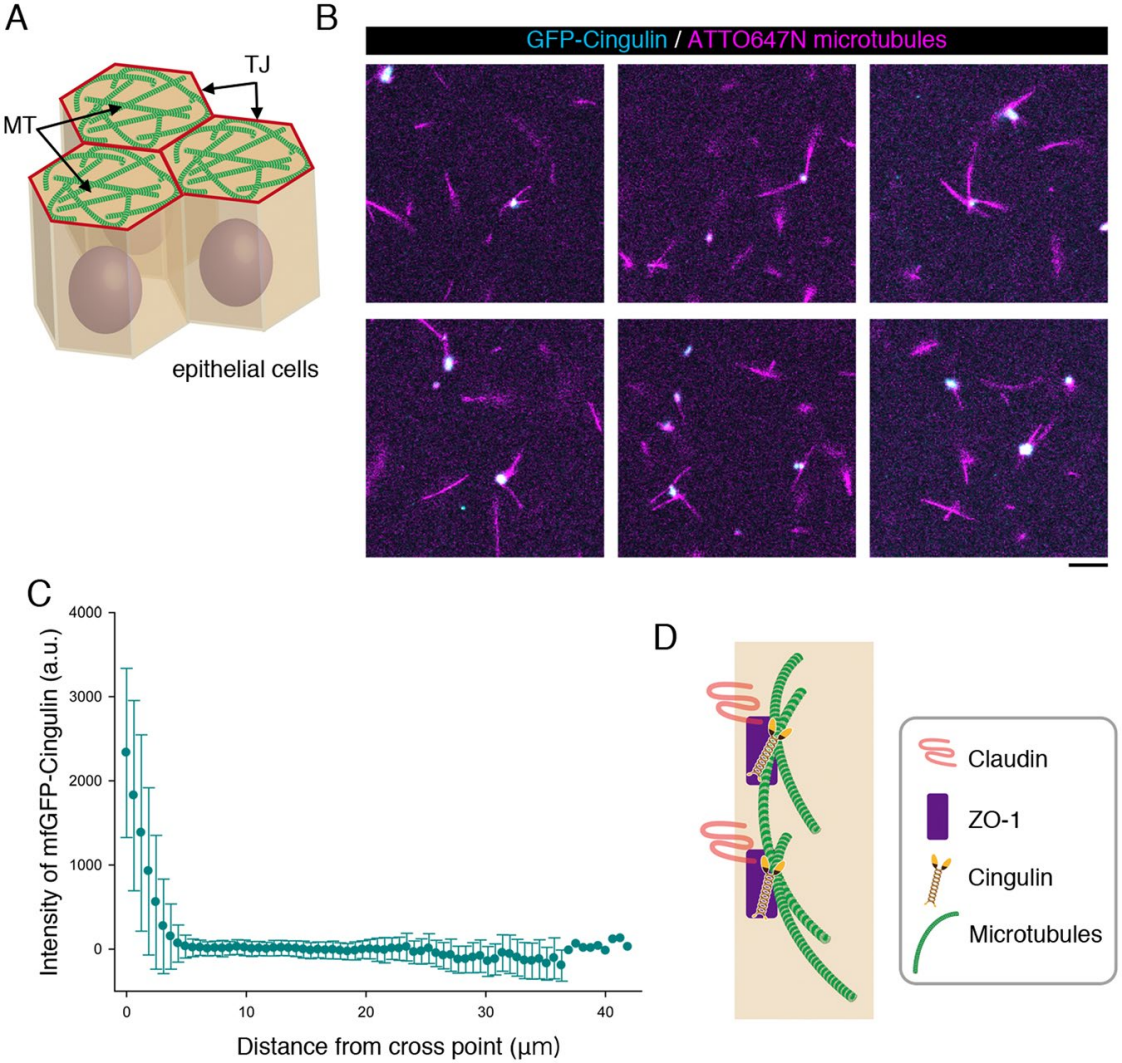
Localization of mfGFP-cingulin to microtubule cross points. (**A**) Schematic drawing of the association of the apical microtubule network with TJs. It appears that the edge of the apical microtubule network associates with TJs in a side-by-side fashion. TJ; tight junciton, MT; microtubules. (**B**) Merged images of ATTO647N-labeled microtubules (magenta) and mfGFP-cingulin (cyan). The concentration of microtubules (tubulin) and mfGFP-cingulin was 10 nM and 50 nM, respectively. Bar indicates 20 μm. (**C**) Graph showing the intensity of mfGFP-cingulin along microtubules (n = 88 from three experiments). Bars indicate standard deviation. The origin of the x-axis was the cross point of microtubules. (**D**) Schematic drawing of the binding pattern of cingulin to microtubules in TJs. Cingulin is anchored to claudin by ZO-1 and localized at cross point of microtubules. Thus, cingulin mediates the side-by-side association of microtubule with TJs.

We previously reported that cingulin mediates the side-by-side association of the apical microtubules network with TJs (Fig. 1A). We also showed that this association was decreased in cingulin knock-down cells^14^. However, the binding characteristics of cinguin to microtubules remained unclear. To investigate the molecular mechanism of cingulin’s binding activity, we first visualized the binding of fluorescently tagged cingulin and microtubules. GFP-tagged cingulin was expressed and purified in Expi293F cells, then mixed with ATTO647N-tagged microtubule filaments. After the mixture was incubated, it was fixed on a cover glass and observed by TIRF microscopy. Interestingly, the images revealed that cingulin molecules (53.2 nM) were localized to the cross points of microtubules (tubulin: 10 nM) (Fig. 1B). Furthermore, to confirm these images, the distance between the microtubule cross points and cingulin molecules was estimated, and indicated that more than 90% of the microtubule-associated cingulin molecules were localized to the cross points of microtubules (Fig. 1C). These reconstitution experiments supported our previous claim that cingulin binds to apical microtubule networks at the sides of microtubules, to cross-link individual microtubules (Fig. 1D).

### Cingulin’s rod 2 domain binds to cingulin’s head domain

**Figure 2:**
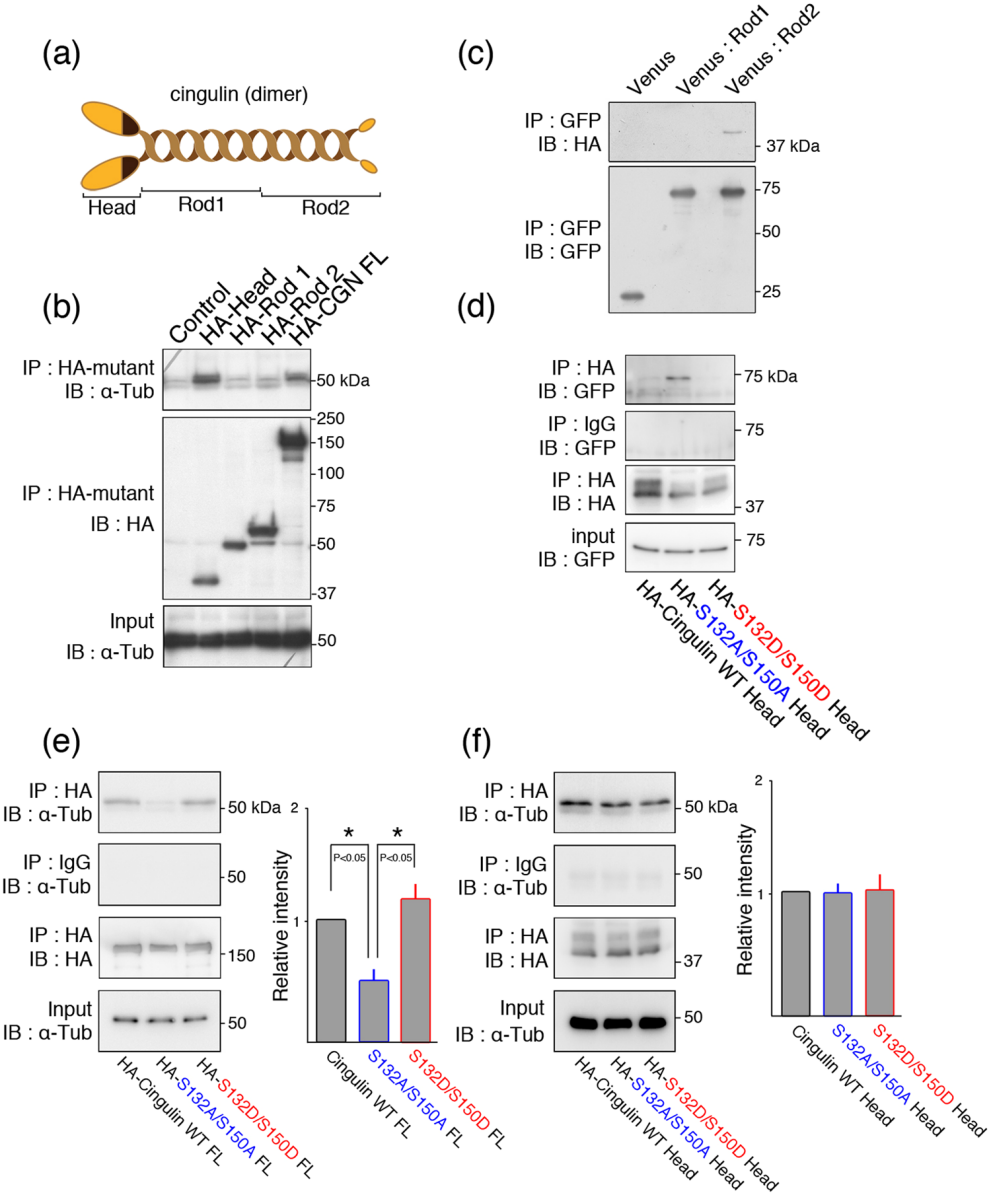
The dephosphorylated head domain of cingulin interacts with its Rod 2 domain. (**a**) Schematic drawing of the cingulin molecule. (**b**) Analysis of the cingulin domains associated with α-tubulin. α-Tubulin bound to the full-length and the head domain of cingulin. (**c**) Coimmunoprecipitation of cingulin’s head domain with its Rod domains. HA-head of cingulin (HA-Head) and Venus-Rod1 or -Rod2 were exogenously overexpressed in HEK293 cells, and extracts were subjected to pull-down assays with an anti-GFP antibody. (**d**) Coimmunoprecipitation of the Rod2 domain of cingulin with a phosphomimetic (S132D/S150D) or dephosphomimetic (S132A/S150A) mutant of the cingulin head domain. HA-mutants of the head domain and Venus-Rod2 were exogenously overexpressed in HEK293 cells, and extracts were subjected to pull-down assays with an anti-GFP antibody. (**e**,**f**) Coimmunoprecipitation of α-tubulin with a phosphomimetic or dephosphomimetic mutant of full-length cingulin (**e**) or its head domain (**f**). HA-full-length (FL) or head domain of cingulin (HA-Head) mutants were exogenously overexpressed in HEK293 cells, and extracts were subjected to pull-down assays with an anti-α-Tubulin antibody. The intensity relative to wild-type (WT) head domain was determined, and the results are expressed as means ± SE (error bars; n = 3). *P < 0.05. IP, Immunoprecipitation. IB, Immunoblot; α-tub, α-tubulin.

It is thought that  cingulin molecules dimerize at their coiled-coil region (Fig. 2a)^26^. The binding of each cingulin domain to endogenous α-tubulin was next examined, using overexpressed HA-tagged constructs of the head, rod 1, and rod 2 domains, as well as full-length cingulin in HEK293 cells. The results showed that both full-length cingulin and its head domain bound to microtubules, with the head domain apparently binding more strongly (Fig. 2b). This finding suggested that some conformational changes might be involved in the binding between α-tubulin and full-length cingulin. To investigate this conformational regulation, the binding between cingulin’s head and rod domains was examined in co-immunoprecipitation assays with the phosphatase inhibitor. The results showed that the head domain was co-immunoprecipitated with an anti-GFP antibody in the mixture containing rod 2 (Fig. 2c), indicating the head domain may interact with the rod 2 domain. We previously reported that α-tubulin binds to cingulin in its phosphorylated form, but not to its dephosphorylated form. We therefore investigated whether AMPK, which phosphorylates head residues serine-132 and -150, is involved in the binding of the head to the rod 2 domain. We also reported that the binding capability of single dephosphorylation mutants of serine-132 (S132A) or -150 residues (S150A) to microtubules was lower than wild-type of cingulin, and the binding capability of double dephosphorylation mutant (S132A/S150A) had the additive effect of their respective dephosphorylation^14^. Hence, we used double phosphorylation or dephosphorylation mutant in this study. Phosphomimetic (S132D/S150D) and dephosphomimetic (S132A/S150A) head mutants were generated and co-transfected with rod 2 into HEK293 cells. The co-immunoprecipitation assay with the phosphatase inhibitor showed that rod 2 bound strongly to head mutants in the dephosphomimetic, but not in the phosphomimetic state (Fig. 2d). We further examined the binding of phosphomimetic and dephosphomimetic forms of full-length cingulin to microtubules (Fig. 2e,f). The full-length dephosphomimetic mutant showed weaker binding to α-tubulin than did the wild-type cingulin or its phosphomimetic mutant (Fig. 2e). On the other hand, dephosphomimetic and phosphomimetic mutants of head domain alone displayed no significant differences in α-tubulin binding (Fig. 2f). Taken together, these observations indicated that self-folding may take place within the cingulin molecule, and that the resulting conformational change may affect the cingulin-microtubule binding.

### The cingulin molecule has two conformations

**Figure 3:**
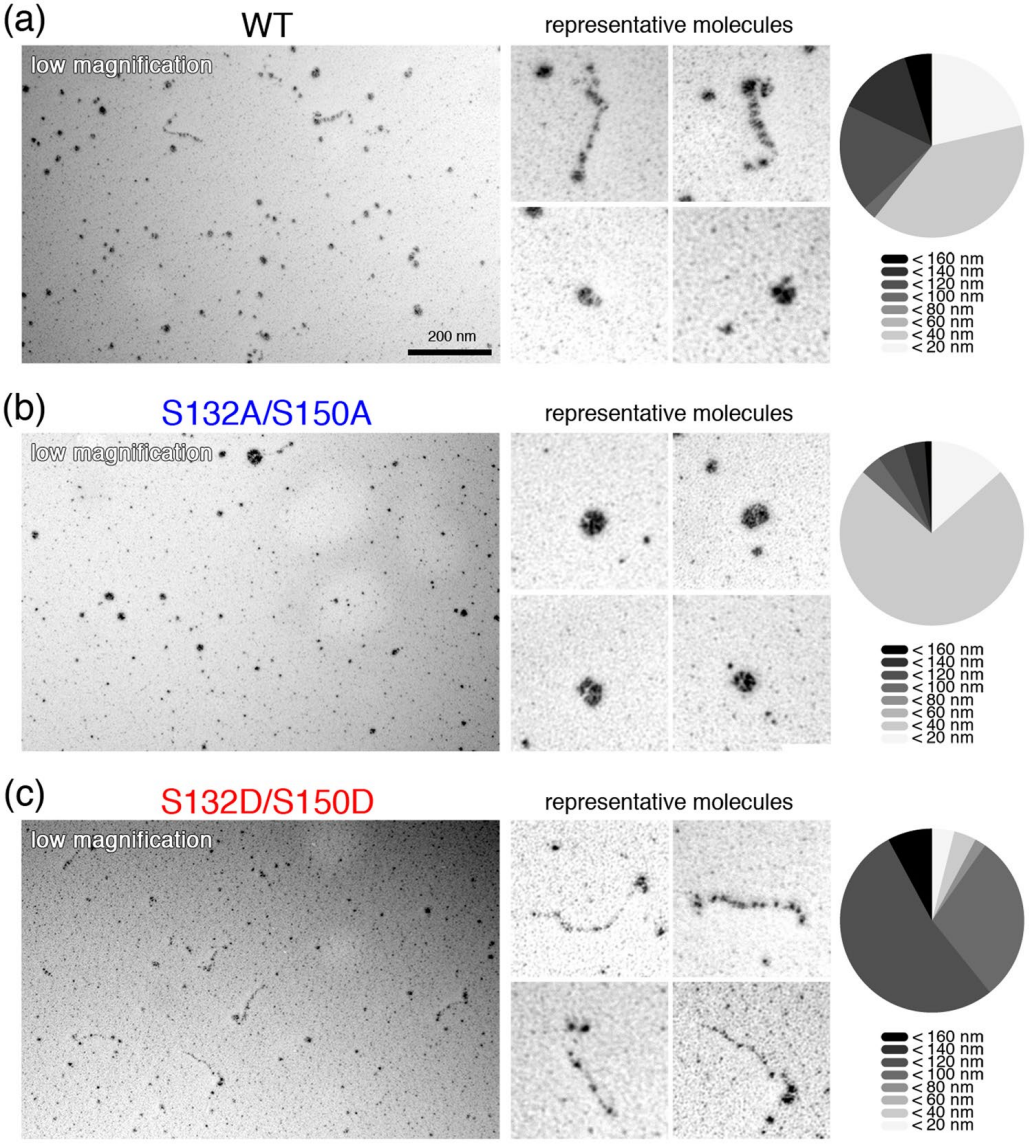
Conformations of wild-type cingulin, and its phosphomimetic (S132D/S150D), and dephosphomimetic (S132A/S150A) mutants. (**a**) Wild-type cingulin proteins have “open” and “closed” conformations. (**b,c**) The dephosphomimetic cingulin mutant formed the “closed” form and the phosphomimetic mutant formed the “open” conformation. Bar, 200 nm.

Electrophoresis of the wild-type, dephosphomimetic and phosphomimetic cingulin purified from baculovirus-infected insect cells in the presence of the phosphatase inhibitor showed clear bands, indicating that it was highly pure (Fig. S1). To visualize the molecules, we subjected the purified cingulin to low-angle rotary-shadowing electron microscopy, which revealed that the cingulin molecules have two distinct shapes: an elongated “open” form, and a shorter “closed” form (Fig. 3a). The open form was structurally similar to myosin’s dimeric form with a globular head. The open form had an approximate length of 130 nm, while the closed form had an approximate diameter of 30 nm (Fig. 3a). Previous reports showed that the lack of a head domain causes chicken cingulin to assume a rod-like shape^26^. This is the first report that full-length cingulin assumes two types of molecular conformation. To determine whether these cingulin conformations were related to its AMPK-dependent phosphorylation, the molecules of the wild-type cingulin and phosphomimetic, and dephosphomimetic cingulin mutants were observed by electron microscopy. The results revealed that, although wild-type cingulin was found in “closed” forms rather than “open” form (Fig. 3a), the dephosphomimetic cingulin mutant tended to be the “closed” form (n = 85) (Fig. 3b), while the molecules of phosphomimetic cingulin mutant take the “open” form more frequently as compared to those of wild-type and dephosphomimetic cingulin (n = 67) (Fig. 3c).

### AMPK phosphorylation is essential for cingulin’s “open” conformation

**Figure 4:**
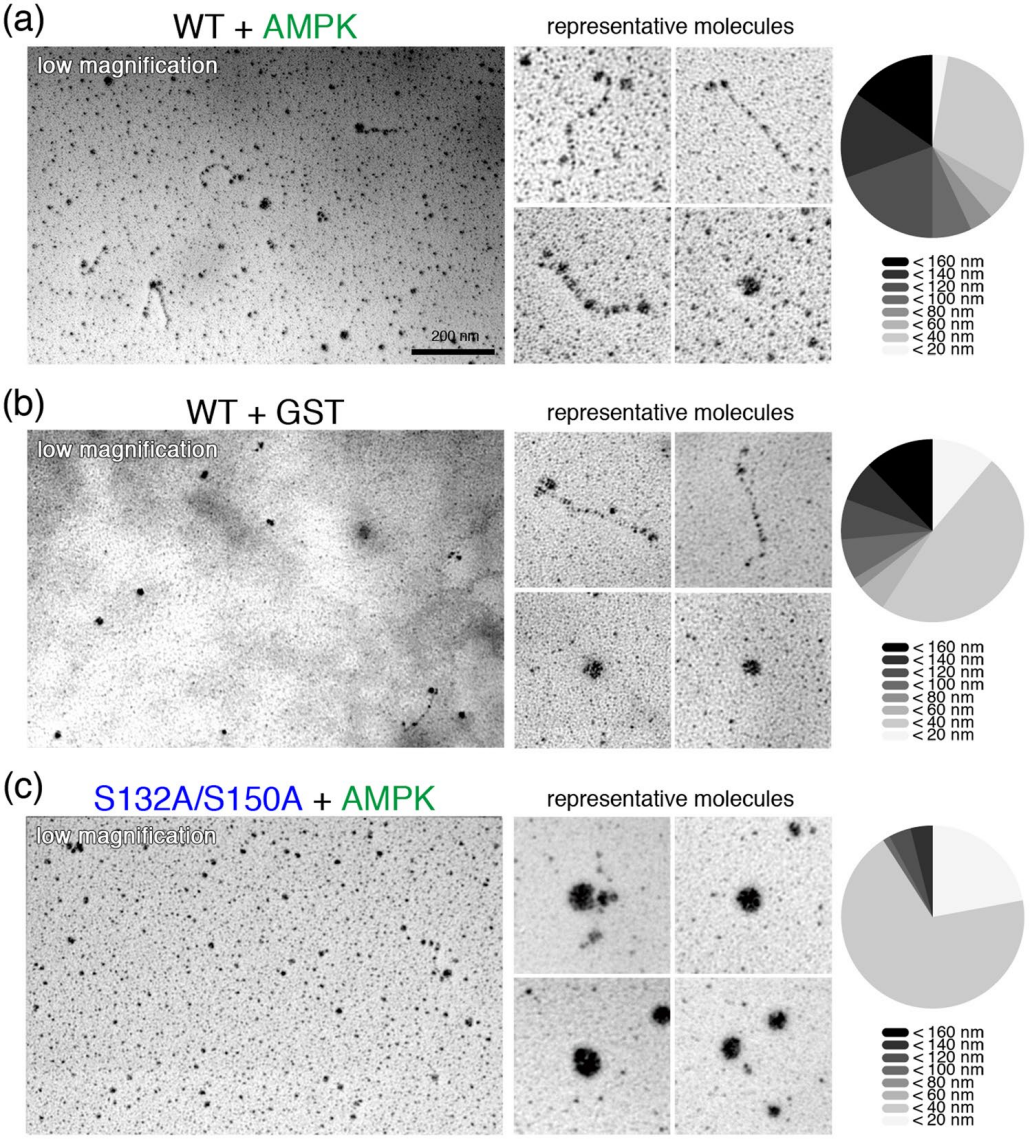
The conformations of cingulin were regulated by AMP-activated protein kinase (AMPK). (**a,b**) AMPK changed the conformation of cingulin protein molecules from “closed” to “open”. (**c**) The conformation of the dephosphomimetic cingulin mutant was not changed by AMPK. Bar, 200 nm.

We reported that the cingulin is the substrate of AMPK by *in vitro* phosphorylation assay^14^. To determine if AMPK phosphorylation regulates cingulin’s molecular conformation, purified wild-type cingulin and its dephosphomimetic mutant were incubated with GST-tagged AMPK *in vitro*, respectively. Each mixture of the two types of cingulin and GST-AMPK α1/β1/γ1 were incubated with ATP at 30 °C for 90 min, and the GST-AMPK was then adsorbed to glutathione sepharose beads. The supernatants were then collected and analyzed by low-angle rotary-shadowing electron microscopy. The results showed that, while 25% of the wild-type cingulin incubated with GST showed the ‘‘open’’ form (n = 83), the percentage rose to 65% or more for wild-type cingulin incubated with GST-AMPK (n = 81) (Fig. 4a,b). On the other hand, the dephosphomimetic cingulin mutant tended to the “closed” form (n = 63), regardless of the presence of AMPK (Fig. 4c).

### AMPK phosphorylation of cingulin’s head domain regulates the binding of cingulin to actin filaments

**Figure 5:**
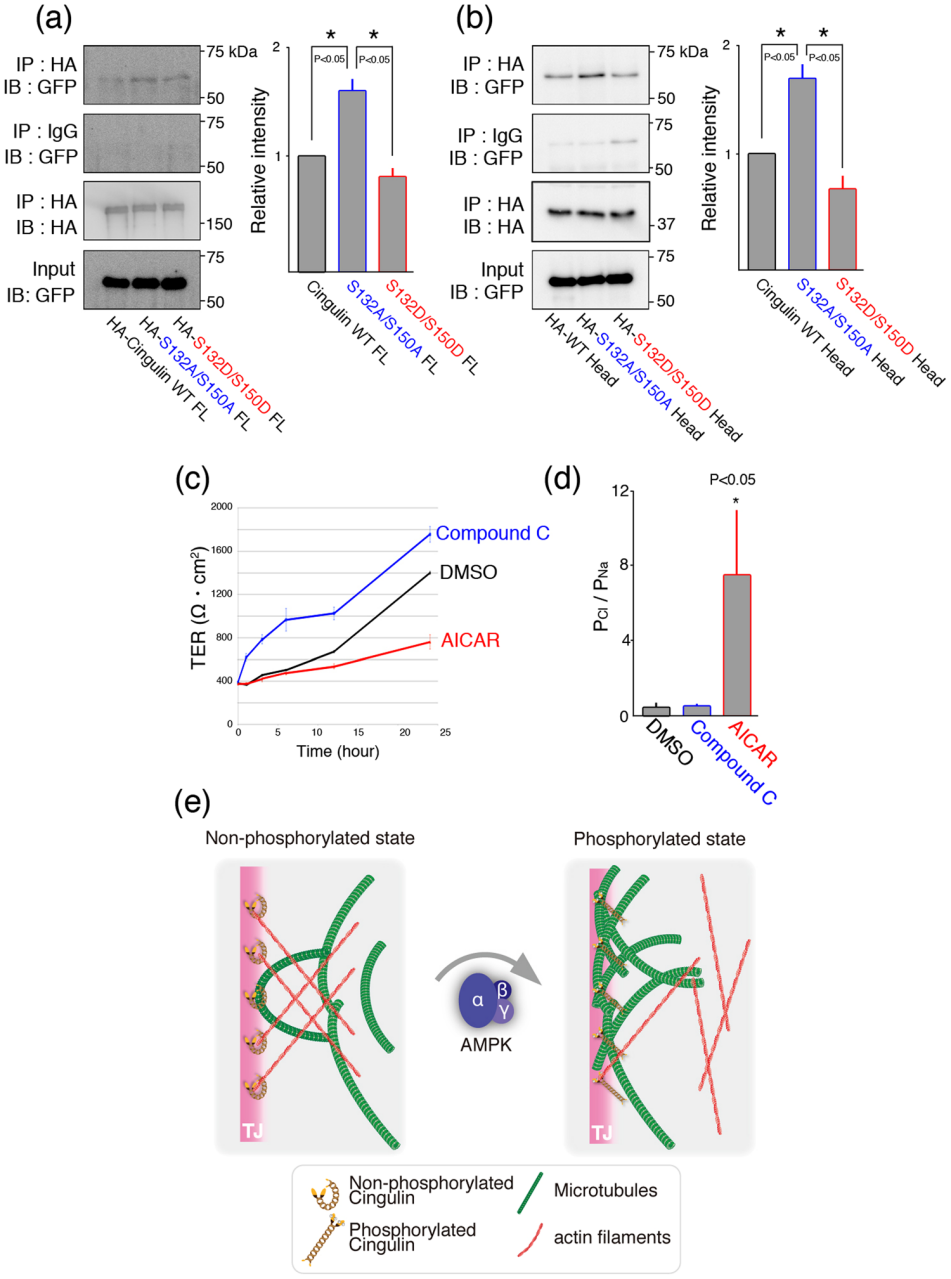
AMPK activity affects the TJ function. (**a,b**) Coimmunoprecipitation of actin with phosphomimetic or dephosphomimetic mutants of full-length cingulin (**a**) or its head domain (**b**). HA-full-length (FL) or head domain of cingulin (HA-Head) mutants and GFP-actin were exogenously overexpressed in HEK293 cells, and extracts were subjected to pull-down assays with an anti-GFP antibody. The band intensities relative to wild type (WT) were determined, and the results are expressed as means ± SE (error bars; n = 3). *P < 0.05. IP, Immunoprecipitation. IB, Immunoblot. (**c**) Measurement of transepithelial electric resistance in control (DMSO) AMPK inhibitor-treated (Compound C; 20 μM), and AMPK activator-treated (AICAR; 2.5 mM) (n = 3/group) epithelia. (**d**) Transepithelial ion permeabilities for Na^+^ and Cl^−^ of DMSO-, Compound C-, and AICAR-treated epithelia. The values were obtained from the dilution potentials, which were calculated using the Goldman–Hodgkin–Katz equation (n = 3). The PCl/PNa values for the DMSO, AICAR-, and Compound C-treated epithelial cells were analyzed statistically. *P < 0.05. (**e**) Schematic drawing of the proposed mechanism for the regulation of cingulin’s binding to two cytoskeletons. The phosphorylation state of cingulin by AMPK reversibly regulates its binding to actin filaments and microtubules.

Cingulin is reported to be an actin-binding protein. We next investigated how AMPK-mediated phosphorylation affects the cingulin-actin binding. When wild-type, dephosphomimetic, or phosphomimetic cingulin was subjected to immunoprecipitation with Venus-tagged actin, the dephosphomimetic form showed a higher interaction with actin than did the others (Fig. 5a). In addition, the wild-type, dephosphomimetic, and phosphomimetic forms of cingulin’s head domain were immunoprecipitated with Venus-tagged actin. The results showed that the dephosphomimetic form of cingulin’s head had the strongest interaction with actin, while the wild-type and phosphomimetic head domains showed a similar, weaker actin binding (Fig. 5b). Collectively, these observations suggested that the actin-binding interaction is lower when cingulin’s head domain is phosphorylated by AMPK. Finally, we investigated the effect of this lower cingulin-actin binding on mouse Eph4 epithelial cell sheet characteristics. First, we measured the TER of the cell sheets over time in the absence or presence of an AMPK inhibitor (compound C) or an AMPK activator (AICAR). The TER increased in the presence of compound C and decreased in the presence of AICAR (Fig. 5c). We next examined the permselectivity of the cell sheet, and found that its permeability for Na^+^ and Cl^−^ ions was increased in the presence of AICAR (Fig. 5d). Taken together, these results indicated that cingulin’s phosphorylation by AMPK decreases its binding to actin filaments, resulting in changed barrier functions of epithelial cell sheets.

## Discussion

Cingulin is a major TJ component. It binds to ZO proteins, which bind to TJ transmembrane proteins, like claudin family members and cytoskeletal components. The TJ includes a variety of scaffolding proteins, including ZO proteins and cingulin, and a number of studies have reported the involvement of these proteins with actomyosin. ZO-1 was discovered as a TJ protein^30^, and was found to contain a PDZ domain^31,32^. Later studies revealed that TJs also include ZO-2 and -3, which have similar molecular domains as ZO-1^33–35^. The ZO proteins are reported to bind actin through their C-terminal domain, and to bind AJ proteins like α-catenin and afadin through their SH3/GUK domains^36,37^. The TJ and AJ function together, within a larger structure known as the AJC. It was reported that in ZO-1 KO/ZO-2 KD Eph4 cells, TJs do not form^38^, and myosin does not become integrated into the AJCs^39^. On the other hand, in a ZO1 KD/ ZO2 KD cell line derived from MDCK cells, myosin was found to be enriched in AJCs^40^. Although the results may vary depending on the cell line, it has been hypothesized that that various cell conditions affect myosin’s integration into the AJC.

After ZO-1, cingulin was discovered as the second TJ protein^24^, and identified as an actin-binding protein^26^. In addition, by binding GEF-H1 and MgcRacGAP, cingulin was shown to regulate myosin phosphorylation via RhoA and Rac1^41,42^. Prekeris and colleagues and we have reported that cingulin also binds microtubules^29^. Furthermore, we discovered a microtubule network underneath the apical membrane of epithelial cell sheets, and reported that the sides of these microtubules associate with TJs via cingulin. In addition, this association is regulated by cingulin’s phosphorylation via AMPK, and its impairment results in anomalies in the formation of epithelial cell colonies^14^. Mangan and others focused on the localization of the apical membrane initiation site (AMIS) during the apical surface formation of proliferating cells, and the regulation of FIP5-endosome targeting. They showed that cingulin’s recruitment of the AMIS is mediated by branched actin formation and cingulin’s direct binding to microtubules^29^. Here we showed that cingulin binds to both actin and microtubules, thereby serving as an important contributor to the epithelial structural formation and characteristics.

It has not been known whether (1) cingulin tends to bind both actin and microtubules, or (2) it has the preference for the binding to either actin or microtubules. The interaction between actin and microtubules has been extensively studied, and found to be important in the structural formation of neural cells, epithelial cells, and fibroblasts^43–46^. Based on these reports, a number of proteins that bind to both actin and microtubules have been described. To understand the function of these proteins, it is important to know whether it is a cross-linker that binds actin and microtubules simultaneously or not. Here we examined whether cingulin’s phosphorylation by AMPK affects its ability to bind microtubules and actin filaments. We found using electron microscopy that cingulin under AMPK phosphorylation undergoes a conformational change that enables it to bind microtubules (Figs 2e,f, 3 and 4). On the other hand, AMPK phosphorylation of the cingulin head weakened its binding to actin (Fig. 5a,b). Thus, cingulin’s binding with microtubules and actin is regulated by AMPK in opposite manners; it follows that the TJ function also changes depending on which filaments are more likely to bind to cingulin (Fig. 5c,d). These results suggest that the binding of de-phosphorylated cingulin by AMPK inhibitor to actin filament cause the well-established TJ . Moreover, the epithelial cells treated with AMPK activator which mimics low energy status of cells increase the paracellular permeability of Na^+^. Since it is widely known that the accumulation of actin filament in TJs affects TER and Na^+^ is a essential factor of glucose absorption, AMPK ingeniously regulates the TJ function to get nutrition by controlling the tendency of binding of cingulin to actin filament or microtubules.

Since AMPK is a key enzyme in energy metabolism, our findings imply that there is a mechanism through which the nutritional condition of a cell can control the TJ function. Cell metabolism is an important topic in the fields of cancer and aging. Our results suggest that the AJC may play roles in cancer formation and aging via metabolic pathways, providing a new perspective from which to study these human conditions.

## Methods

### Preparation of recombinant cingulin protein

Recombinant cingulin protein fused with multifunctional GFP was purified as previously described^47^. The sequences of full-length mouse cingulin (NM_001037711) and of mfGFP were inserted tandemly into the pcDNA5/FRT/TO vector by the In-Fusion method (Clontech, Palo Alto, California, USA). Expi293F cells (Invitrogen) transfected with pcDNA5/FRT/TO-mfGFP-cingulin were collected by centrifugation at 700 g for 5 min, washed twice with PBS buffer (pH 7.4), and stored at −80 °C. Frozen cells were suspended in lysis buffer [50 mM Tris-HCl pH 7.5, 200 mM NaCl, 1 mM MgSO_4_, 10% (w/v) sucrose, 1 mM dithiothreitol] containing a protease inhibitor cocktail (Nacalai Tesque, Kyoto, Japan). The resuspended cells were then homogenized using a motor-driven stirrer (Three-one-motor, Heidon, Tokyo, Japan). After ultracentrifugation at 109,372 g for 20 min at 4 °C, the supernatant was filtered through a 0.45-μm SFCA filter, loaded onto Strep-Tactin resin (IBA, Göttingen, Germany), and eluted with 50 mM Tris-HCl pH 7.5, 200 mM NaCl, 1 mM MgSO_4_, 10% (w/v) sucrose, and 1 mM dithiothreitol. Protein concentration was determined by the Bradford method with the Coomassie Plus Protein Assay Reagent (Thermo Fisher Scientific). To obtain purified cingulin, recombinant cingulin fused with GST was expressed in a baculovirus-infected insect cell system and purified using Glutathione Sepharose 4B. After purification, this fusion protein was treated with PreScission Protease to cut the GST tag off the cingulin molecule.

### Preparation of tubulin

Tubulin was purified from porcine brain tissue through 2 successive cycles of polymerization and depolymerization in high-molarity PIPES buffer^48^, which removes contaminating microtubule- associated proteins. To prepare fluorescently labeled microtubules, tubulin was labeled with ATTO647N (ATTO-TEC). ATTO647N-labeled microtubules were generated by copolymerizing labeled and unlabeled tubulin for 30 min at 37 °C and were stabilized with 20 µM paclitaxel (Sigma). The labeling ratio was 6%.

### TIRF microscopy and quantification of cingulin on microtubules

The interaction between microtubules and cingulin was observed with a Nikon Ti-E inverted microscope and A1 confocal imaging system as described previously^49^. The observation chamber was composed of 2 coverslips (32 mm × 24 mm and 18 mm × 24 mm) that were coated with Teflon^50^, and spaced with double-sided tape (80-μm thick, W-12, 3 M). The chamber was first passivated using 1% (w/v) Pluronic F127 dissolved in BRB80 (80 mM PIPES-KOH, 1 mM MgSO_4_, 1 mM EGTA) buffer and incubated for 5 min to prevent the nonspecific adsorption of proteins. After washing the chamber with BRB80 buffer supplemented with 10 μM taxol, a mixture of fluorescently labeled microtubules and mfGFP-cingulin in assay buffer (80 mM PIPES-KOH pH 6.8, 2 mM MgSO_4_, 1 mM EGTA, 2 mM dithiothreitol, 42.5 U/mL glucose oxidase [Sigma], 42.5 U/mL catalase, 10 μM taxol, 25 mM glucose) was applied to the chamber. To quantify the mfGFP-cingulin at microtubule cross points, we measured the intensity of mfGFP-cingulin along microtubules with the origin at the cross points. Before calculating the mean value, the background value was subtracted from the raw value.

### Reagents

The primary antibodies were mouse anti-α-tubulin monoclonal antibody (mAb) (Sigma-Aldrich), rat anti-α-tubulin mAb (Abcam), mouse anti-HA mAb (Covance), rat anti-HA mAb (Roche), and rat anti-GFP mAb (Nacalai Tesque) antibodies. Mouse anti-cingulin mAb and was gifted by K. Owaribe (Nagoya University, Nagoya, Japan) and rat anti-cingulin was kindly provided by M. Furuse (National Institute for Physiological Sciences, Okazaki, Japan). HRP-conjugated secondary antibodies were also purchased (BD). Phosphatase inhibitor cocktail tablets and protease inhibitor cocktail are purchased (Roche, Nacalai tesque). Compound C and AICAR were purchased (EMD Millipore, Sigma).

### Cell culture and transfection

Mouse Eph4 epithelial cells and HEK293 cells were grown in Dulbecco’s modified Eagle’s medium supplemented with 10% fetal calf serum. Transfection was performed using Lipofectamine Plus reagent (Invitrogen) according to the manufacturer’s instructions.

### Immunoprecipitation

HEK293 cells were transfected with expression vectors. Cell lysates were incubated with protein A–Sepharose bound with the anti-α-tubulin or anti-HA antibody. Immune complexes were fully washed and then resuspended in 40 μl SDS sample buffer, and 5- and 20-μl aliquots of each were analyzed by Western blotting.

### Western blotting

The protein samples were separated by SDS-PAGE, transferred onto a PVDF membrane, and blotted with the appropriate antibodies. To quantify the signals in Western blots, densitometry of the immunoblot bands compared with a loading control in the same membrane was performed using ImageJ software (National Institutes of Health).

### Cingulin phosphorylation assay

Cingulin phosphorylation assays were performed at 30 °C in a reaction volume of 30 μl containing 20 mM Tris-HCl, pH 7.4, 0.3 mM NaCl, 0.2 mM AMP, 0.8 mM MgCl_2_, and 0.2 mM ATP, 0.1 mM recombinant AMPK α1/β1/γ1 (Carna Biosciences), and 1 μg of cingulin or cingulin mutants.

### Low-angle rotary-shadowing electron microscopy

The molecular shape of purified cingulin and cingulin mutants, with or without incubation with AMPK, was analyzed by low-angle rotary-shadowing electron microscopy^51^. After adding equal amounts of glycerol to the cingulin samples, they were splayed on freshly split mica. The droplets were dried in a vacuum, and sprinkled with platinum and carbon from above. The samples were then observed by an electron microscope (JEM-1400; JEOL).

### Transepithelial resistance (TER) and electrophysiology measurements

Aliquots of 1 × 10^5^ Eph4 cells were plated on a 12-mm-diameter transwell insert, and the TER was measured directly in the culture medium using a Millicell-ERS epithelial volt-ohmmeter (Merck Millipore).

To characterize the epithelia electrophysiologically, the transwell filters were mounted in Ussing chambers. The chambers were filled with 5 ml of solution (150 mM NaCl, 2 mM CaCl_2_, 1 mM MgCl_2_, 10 mM mannitol, and 10 mM Tris-HCl, pH 7.4) and the temperature of the solution in both chambers was maintained at 37 °C with 100% O_2_ bubbling. The transepithelial potential was measured through 3 M KCl-agar bridges connected to calomel electrodes using a voltage-clamping device (Nihon Kohden). To determine the NaCl dilution potential, the basal solution was replaced with one containing 75 mM NaCl instead of 150 mM NaCl. The osmolarity of the solutions was maintained using mannitol.

## Electronic supplementary material

Supplementary figure1, Supplementary figure2, Supplementary figure3, Supplementary figure 4, Supplementary figure 5
