# Publisher Correction: AMPK-dependent phosphorylation of cingulin reversibly regulates its binding to actin filaments and microtubules

**DOI:** 10.1038/s41598-018-37079-4

**Published:** 2018-12-17

**Authors:** Tomoki Yano, Takayuki Torisawa, Kazuhiro Oiwa, Sachiko Tsukita

**Affiliations:** 1 Laboratory of Biological Science, Graduate School of Frontier Biosciences and Graduate School of Medicine, Osaka University, Osaka, 565-0871 Japan; 2 National Institute of Information and Communications Technology, Advanced ICT Research Institute, Kobe, Hyogo, 651-2492 Japan; 3 Present Address: Department of Genetics, School of Life Science, SOKENDAI (The Graduate University for Advanced Studies), Mishima, 411-8540 Japan

Correction to: *Scientific Reports* 10.1038/s41598-018-33418-7, published online 19 October 2018

The original version of this Article contained errors.

In the Introduction,

“Epithelial cell sheets compartmentalize our body, with their apical membranes facing outer environments. To adapt to the ambient outer environment and maintain inner homeostasis, apical membranes develop special architectures that include cytoskeletons^1,2,3,4,5^.”

now reads:

“Epithelial cell sheets compartmentalize our body, with their apical membranes facing outer environments. To adapt to the outer environment and maintain inner homeostasis, apical membranes develop special architectures that include cytoskeletons^1,2,3,4,5^.”

“Cingulin, a TJ protein that forms a complex with one of the zonula occludens proteins (ZO-1, 2, or 3), is an a classical actin-binding protein whose function has been explored^24,25,26,27,28^. Cingulin regulates claudin-2 expression and the G1/S transition via GEF-H1-RhoA activity in Madin-Darby canine kidney cells^29^. Cingulin is composed of three domains: a globular head, a coiled-coil rod, and a small globular tail^30^. The Since the N-terminal globular head domain of cingulin has a high binding affinity for ZO proteins and actin filaments^26,27^, cingulin has been considered an actin-binding protein. On the other hand However, we previously reported that cingulin binds to microtubules and the sides of the microtubules beneath the apical membrane can associate with TJs, and at these sites, cingulin the sides of the microtubules beneath the apical membrane can associate with TJs, and at these sites, cingulin is phosphorylated in its head domain by AMP-activated protein kinase (AMPK) tethered to the microtubules^14^. Recent studies have shown that cingulin directly binds to the C-terminal domain of tubulin an interaction between actin and tubulin promotes cingulin recruitment during establishment of the apical membrane initiation site (AIMS) and formation of the apical lumen^30^. However, the dynamic aspects of cingulin’s binding to actin filaments and microtubules, and the relative binding affinities of cingulin to actin filaments and microtubules remain unclear.”

now reads:

“Cingulin, a TJ protein that forms a complex with one of the zonula occludens proteins (ZO-1, 2, or 3), is an a classical actin-binding protein whose function has been explored^24,25,26,27,28^. Cingulin is composed of three domains: a globular head, a coiled-coil rod, and a small globular tail^30^. The N-terminal globular head domain of cingulin has a high binding affinity for ZO proteins and actin filaments^26,27^. On the other hand, we previously reported that cingulin binds to microtubules and is phosphorylated in its head domain by AMP-activated protein kinase (AMPK)^14^. Recent studies have shown that cingulin directly binds to the C-terminal domain of tubulin^30^. However, the dynamic aspects of cingulin’s binding to actin filaments and microtubules, and the relative binding affinities of cingulin to actin filaments and microtubules remain unclear.”

“We discovered that the phosphorylated cingulin head domain tends to detach from actin filaments, and this tendency effects on affects the TJ’s permselectivity and sealing properties.”

now reads:

“We discovered that the phosphorylated cingulin head domain tends to detach from actin filaments, and this tendency effects on the TJ’s permselectivity and sealing properties.”

In the Results section under subheading ‘Cingulin is a cross-linker of microtubules’,

“We previously reported that cingulin mediates the side-by-side association of the apical microtubules network with TJs beneath of the apical membrane of epithelial cells (Fig. 1A).”

now reads:

“We previously reported that cingulin mediates the side-by-side association of the apical microtubules network with TJs (Fig. 1A).”

In the Results section under subheading ‘Cingulin’s rod 2 domain binds to cingulin’s head domain’,

“To investigate this conformational regulation, the binding between cingulin’s head and rod domains was examined in co-immunoprecipitation assays with the phosphatase inhibitor of HA-head and venus-rod 1/2 domains in HEK293 cells.”

now reads:

“To investigate this conformational regulation, the binding between cingulin’s head and rod domains was examined in co-immunoprecipitation assays with the phosphatase inhibitor.”

In the Results section under subheading ‘The cingulin molecule has two conformations’,

“The results revealed that, although wild-type cingulin was found in “closed” forms than “open” form (Fig. 3a), the dephosphomimetic cingulin mutant tended to be the “closed” form (n = 85) (Fig. 3b), while the molecules of phosphomimetic cingulin mutant take the “open” form more frequently as compared to those of wild-type and dephosphomimetic cingulin (n = 67) (Fig. 3c).”

now reads:

“The results revealed that, although wild-type cingulin was found in “closed” forms rather than “open” form (Fig. 3a), the dephosphomimetic cingulin mutant tended to be the “closed” form (n = 85) (Fig. 3b), while the molecules of phosphomimetic cingulin mutant take the “open” form more frequently as compared to those of wild-type and dephosphomimetic cingulin (n = 67) (Fig. 3c).”

In the Results section under subheading ‘AMPK phosphorylation is essential for cingulin’s “open” conformation’,

“Each Mixtures of the two types of cingulin and GST-AMPK α1/β1/γ1 were incubated with ATP at 30 °C for 90 min, and the GST-AMPK was then adsorbed to glutathione sepharose beads. The supernatants were then collected and analyzed by low-angle rotary-shadowing electron microscopy. The results showed that, while 25% of the wild-type cingulin incubated with GST showed the ‘‘open’’ form (n=83), the percentage rose to 65% or more for wild-type cingulin incubated with GST-AMPK (n=81) the molecules of wild-type cingulin incubated with AMPK frequently came into the “open” form (n = 81) (Fig. 4a,b) as compared with the molecules of wild-type cingulin incubated with GST (n = 83) (Fig. 4b).”

now reads:

“Each mixture of the two types of cingulin and GST-AMPK α1/β1/γ1 were incubated with ATP at 30 °C for 90 min, and the GST-AMPK was then adsorbed to glutathione sepharose beads. The supernatants were then collected and analyzed by low-angle rotary-shadowing electron microscopy. The results showed that, while 25% of the wild-type cingulin incubated with GST showed the ‘‘open’’ form (n=83), the percentage rose to 65% or more for wild-type cingulin incubated with GST-AMPK (n=81) (Fig. 4a,b).”

In the Results section under subheading ‘AMPK phosphorylation of cingulin’s head domain regulates the binding of cingulin to actin filaments’,

“Cingulin is reported to be an actin-binding protein. We next investigated how AMPK-mediated phosphorylation affects the cingulin-actin binding under blocking phosphatases condition.”

now reads:

“Cingulin is reported to be an actin-binding protein. We next investigated how AMPK-mediated phosphorylation affects the cingulin-actin binding.”

In the Discussion section,

“Cingulin is a major TJs component. It binds to ZO proteins, which also bind to other TJs transmembrane proteins, like claudin family members and cytoskeletal components. The TJ includes a variety of scaffolding proteins, including ZO proteins and cingulin, and a number of studies have reported the involvement of these proteins with actomyosin. ZO-1 was discovered as a TJ protein^31^, and was found to contain a PDZ domain^32,33^. Later studies revealed that TJs also include ZO-2 and -3, which have similar molecular domains as ZO-1^34,35,36^. The ZO proteins are reported to bind actin through their C-terminal domain, and to bind AJ proteins like α-catenin and afadin Afadin through their SH3/GUK domains^37,38^. The TJ and AJ function closely together, within a larger structure known as the AJC. It was reported that in ZO-1 KO/ZO-2 KD cells, TJs do not form^39^, and myosin does not become integrated into the AJCs^40^. On the other hand, in a ZO1 KD/ZO2 KD cell line derived from MDCK cells, the myosin AJCs was were found to be enriched in AJCs myosin^41^. Although the results may vary depending on the cell line, it has been hypothesized that that various cell conditions affect myosin’s integration into the AJC.”

now reads:

“Cingulin is a major TJ component. It binds to ZO proteins, which bind to TJ transmembrane proteins, like claudin family members and cytoskeletal components. The TJ includes a variety of scaffolding proteins, including ZO proteins and cingulin, and a number of studies have reported the involvement of these proteins with actomyosin. ZO-1 was discovered as a TJ protein^31^, and was found to contain a PDZ domain^32,33^. Later studies revealed that TJs also include ZO-2 and -3, which have similar molecular domains as ZO-1^34,35,36^. The ZO proteins are reported to bind actin through their C-terminal domain, and to bind AJ proteins like α-catenin and afadin through their SH3/GUK domains^37,38^. The TJ and AJ function together, within a larger structure known as the AJC. It was reported that in ZO-1 KO/ZO-2 KD Eph4 cells, TJs do not form^39^, and myosin does not become integrated into the AJCs^40^. On the other hand, in a ZO1 KD/ZO2 KD cell line derived from MDCK cells, myosin was found to be enriched in AJCs^41^. Although the results may vary depending on the cell line, it has been hypothesized that that various cell conditions affect myosin’s integration into the AJC.”

“After ZO-1, cingulin was discovered as the second TJs protein to be the second TJs protein to be discovered^24^, and like ZO-1 was known and identified as an actin-binding protein^26^. In addition, by binding GEF-H1 and MgcRacGAP, cingulin was shown to regulate myosin phosphorylation via RohA and Rac1^29,42^. Prekeris and colleagues and we have reported that cingulin also binds microtubules^30^. Furthermore, we discovered a microtubule network underneath the apical membrane of epithelial cell sheets, and reported that the sides of these microtubules associate with TJs via cingulin. In addition, this association takes part in cingulin’s phosphorylation via AMPK, and its impairment results in anomalies in the formation of epithelial cell colonies^14^.”

now reads:

“After ZO-1, cingulin was discovered as the second TJ protein ^24^, and identified as an actin-binding protein^26^. In addition, by binding GEF-H1 and MgcRacGAP, cingulin was shown to regulate myosin phosphorylation via RhoA and Rac1^29,42^. Prekeris and colleagues and we have reported that cingulin also binds microtubules^30^. Furthermore, we discovered a microtubule network underneath the apical membrane of epithelial cell sheets, and reported that the sides of these microtubules associate with TJs via cingulin. In addition, this association is regulated by cingulin’s phosphorylation via AMPK, and its impairment results in anomalies in the formation of epithelial cell colonies^14^.”

“We found using electron microscopy that cingulin under AMPK phosphorylation undergoes a conformational change that which enables it to bind microtubules (Figs 2e,f, 3 and 4). On the other hand, AMPK phosphorylation of the cingulin head weakened its binding to actin (Fig. 5a,b). Thus, cingulin’s binding with microtubules and actin is regulated by AMPK in opposite manners; it follows that the TJ function also changes depending on which filaments are more likely to bind to cingulin (Fig. 5c,d). These results suggest that the binding of de-phosphorylated cingulin by AMPK inhibitor to actin filament cause the well-established TJ tight junction. Moreover, the epithelial cells treated with AMPK activator which mimics low energy status of cells increase the paracellular permeability of Na^+^. From the view points that it is widely known that the accumulation of actin filament in TJs affects TER and Na^+^ is a essential factor of glucose absorption, AMPK ingeniously regulates the TJ function to get nutrition by controlling the tendency of binding of cingulin to actin filament or microtubules.”

now reads:

“We found using electron microscopy that cingulin under AMPK phosphorylation undergoes a conformational change that enables it to bind microtubules (Figs 2e,f, 3 and 4). On the other hand, AMPK phosphorylation of the cingulin head weakened its binding to actin (Fig. 5a,b). Thus, cingulin’s binding with microtubules and actin is regulated by AMPK in opposite manners; it follows that the TJ function also changes depending on which filaments are more likely to bind to cingulin (Fig. 5c,d). These results suggest that the binding of de-phosphorylated cingulin by AMPK inhibitor to actin filament cause the well-established TJ. Moreover, the epithelial cells treated with AMPK activator which mimics low energy status of cells increase the paracellular permeability of Na^+^. Since it is widely known that the accumulation of actin filament in TJs affects TER and Na^+^ is a essential factor of glucose absorption, AMPK ingeniously regulates the TJ function to get nutrition by controlling the tendency of binding of cingulin to actin filament or microtubules.”

Additionally, in Figure 1D the symbol for Microtubules was omitted from the key.

Finally, in the Acknowledgements section,

“We are grateful to Drs K. Owaribe and M. Furuse (National Institute for Physiological Sciences) for the generous gift of the mouse or rat anti-cingulin mAb, respectively. We thank Dr. Uji, Mrs. Takenaga, and Mrs Hagiwara-Yano for technical assistance and members of our laboratories for discussion. We thank graduate student Anna Cho for reading the manuscript. This work was supported in part by Grants-in-Aid for Scientific Research (A) and Creative Scientific Research from the Ministry of Education, Culture, Sports, Science and Technology of Japan (MEXT) (to S.T.) and from CREST (Core Research for Evolutional Science and Technology) of the Japan Science and Technology Agency (JST) (to S.T. and K.O.) This research was also supported by Grants-in-Aid for Young Scientists (B) (to T.Y.).”

now reads:

“We are grateful to Drs K. Owaribe and M. Furuse (National Institute for Physiological Sciences) for the generous gift of the mouse or rat anti-cingulin mAb, respectively. We thank Dr Uji, Ms. Takenaga, and Mrs Hagiwara-Yano for technical assistance and laboratory members for discussion. We thank graduate student Anna Cho for reading the manuscript. This work was supported in part by Grants-in-Aid for Scientific Research (A) and Creative Scientific Research from the Ministry of Education, Culture, Sports, Science and Technology of Japan (MEXT) (to S.T.) and from CREST (Core Research for Evolutional Science and Technology) of the Japan Science and Technology Agency (JST) (to S.T. and K.O.) This research was also supported by Grants-in-Aid for Young Scientists (B) (to T.Y.).”

These errors have now been corrected in the HTML and PDF versions of the original Article.

