# Supplementary material for: AMPK-dependent phosphorylation of cingulin reversibly regulates its binding to actin filaments and microtubules
Source: Sci Rep. 2018 Oct 19;8:15550. doi: 10.1038/s41598-018-33418-7 (PMC6195624; doi:10.1038/s41598-018-33418-7)
Supplement: Supplementary file 1 — Supplementary figure1, Supplementary figure2, Supplementary figure3, Supplementary figure 4, Supplementary figure 5 [file 41598_2018_33418_MOESM1_ESM.pdf]

AMPK-dependent phosphorylation of cingulin reversibly regulates its binding to actin filaments and microtubules

Tomoki Yano[1], Takayuki Torisawa[2], Kazuhiro Oiwa[2], and Sachiko Tsukita[1]

[1] Laboratory of Biological Science, Graduate School of Frontier Biosciences and Graduate School of Medicine, Osaka University, Osaka 565-0871, Japan.

[2] National Institute of Information and Communications Technology, Advanced ICT Research Institute, Kobe, Hyogo 651-2492, Japan

Running title: AMPK regulates the TJ function.

Key words: tight junction, cingulin, microtubules, actin filaments, AMP-activated protein kinase.

Corresponding author: **Sachiko Tsukita**

Laboratory of Biological Science, Graduate School of Frontier Biosciences and Graduate School of Medicine, Osaka University,

2-2 Yamadaoka, Suita, Osaka 565-0871, Japan.

Tel. 81-6-6879-3320; Fax. 81-6-6879-3329

e-mail: atsukita@biosci.med.osaka-u.ac.jp

Competing Interests: None

Supplementary figure legends

Figure S1. (A) Purification of Wild type, phosphorylated mutant, and dephosphorylated cingulin molecules.

Figure S2. Full-length images of the cropped blots presented in the main figures. Full-length images of Figure 2B, C, and D.

Figure S3. Full-length images of the cropped blots presented in the main figures. Full-length images of Figure 2E and F.

Figure S4. Full-length images of the cropped blots presented in the main figures. Full-length images of Figure 5A.

Figure S5. Full-length images of the cropped blots presented in the main figures. Full-length images of Figure 5B.

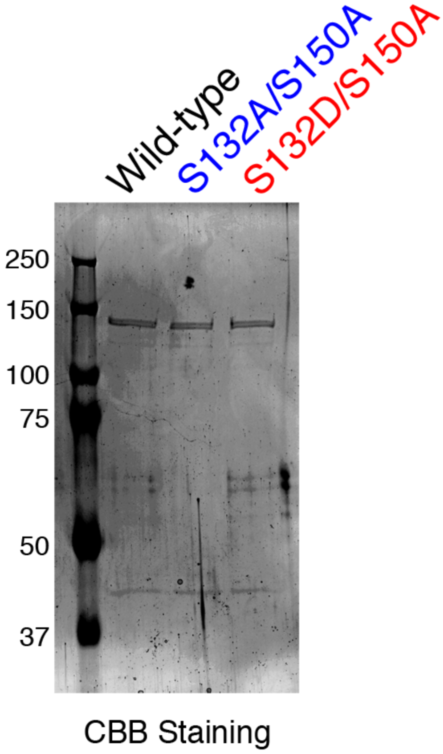

Supplemental Figure S1. Tsukita

Figure 2 B

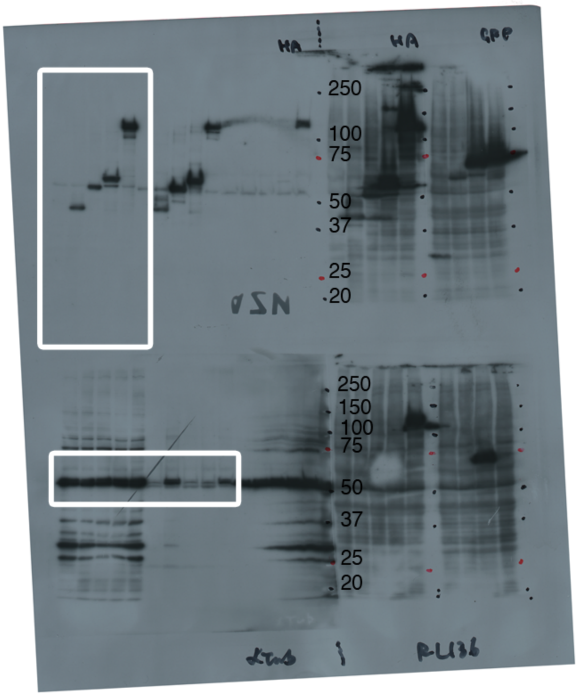

Figure 2 C

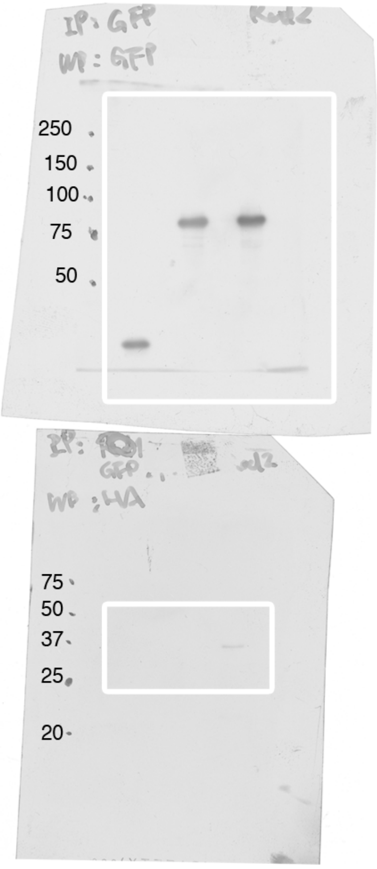

Figure 2 D

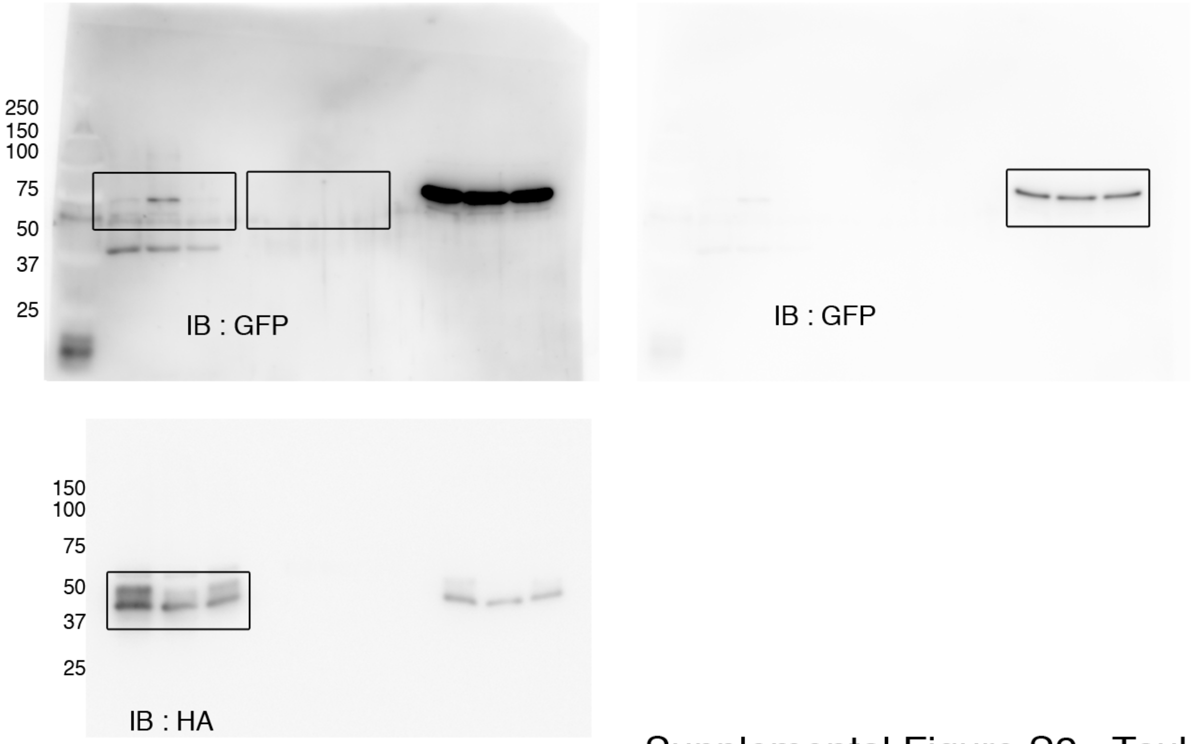

Supplemental Figure S2. Tsukita

Figure 2 E

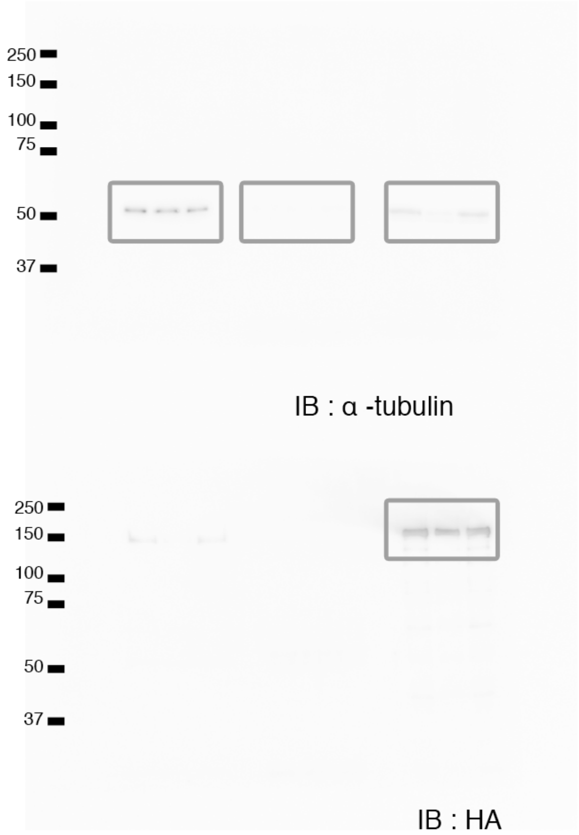

Figure 2 F

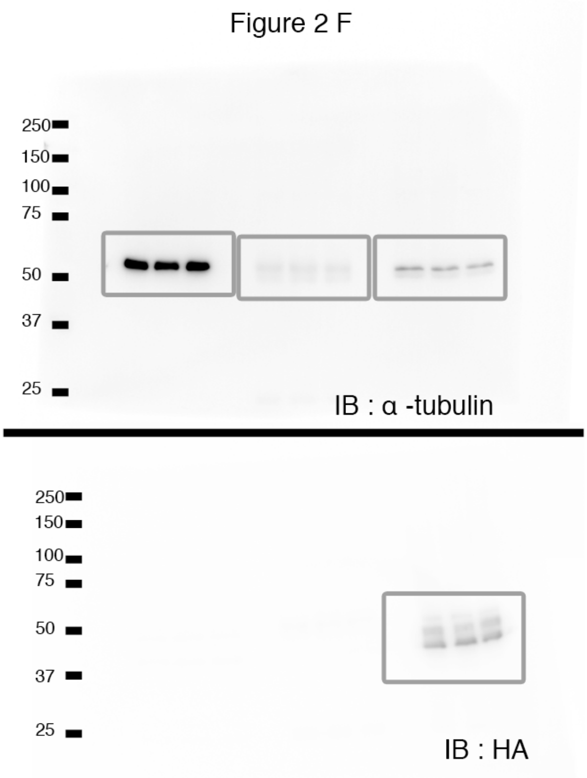

Supplemental Figure S3. Tsukita

Figure 5 A

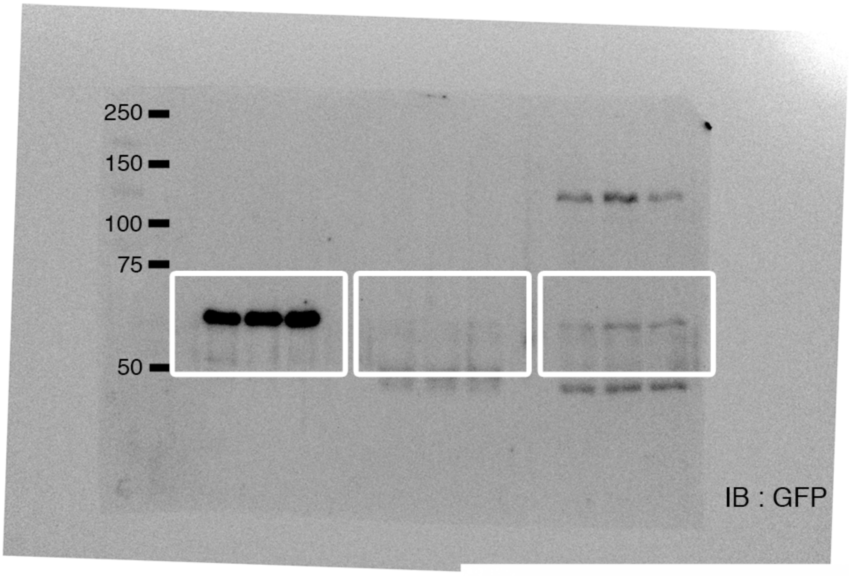

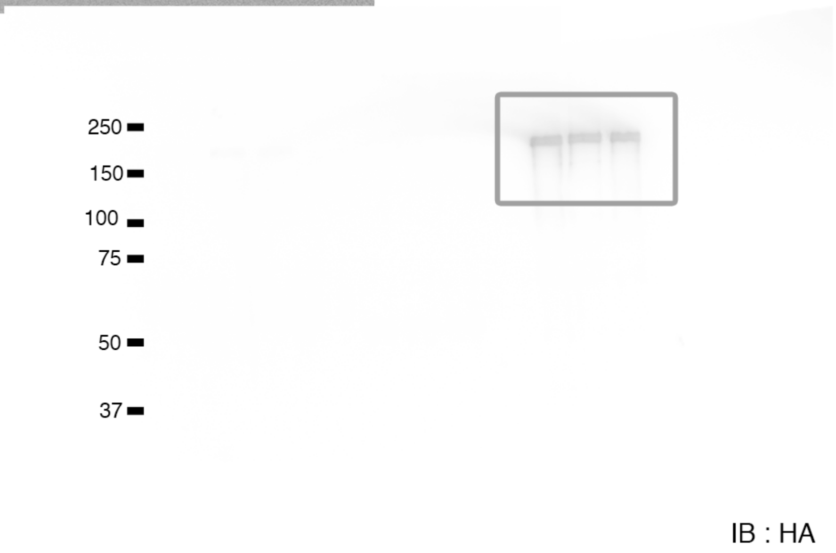

Supplemental Figure S4. Tsukita

Figure 5 B

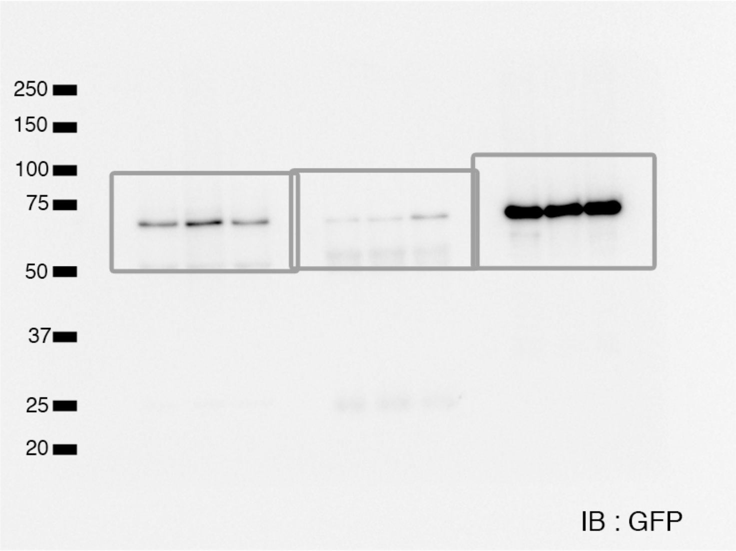

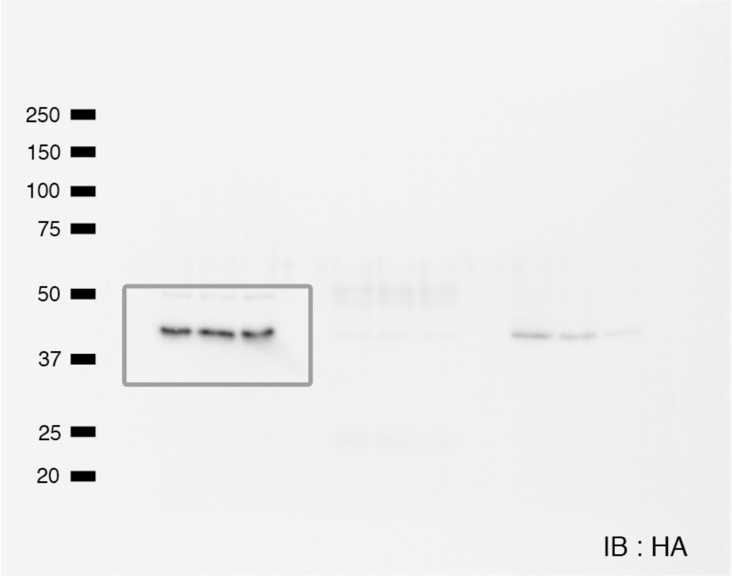

Supplemental Figure S5. Tsukita
